# Supplementary material for: Causal Relationship between Aging and Anorexia Nervosa: A White-Matter-Microstructure-Mediated Mendelian Randomization Analysis
Source: Biomedicines. 2024 Aug 16;12(8):1874. doi: 10.3390/biomedicines12081874 (PMC11351342; doi:10.3390/biomedicines12081874)
Supplement: Supplementary file 1 [file biomedicines-12-01874-s001.zip › Supplementary Figure/Figure S4/Singe SNP plot of MR analysis for TL to ubm-b-2099.pdf]

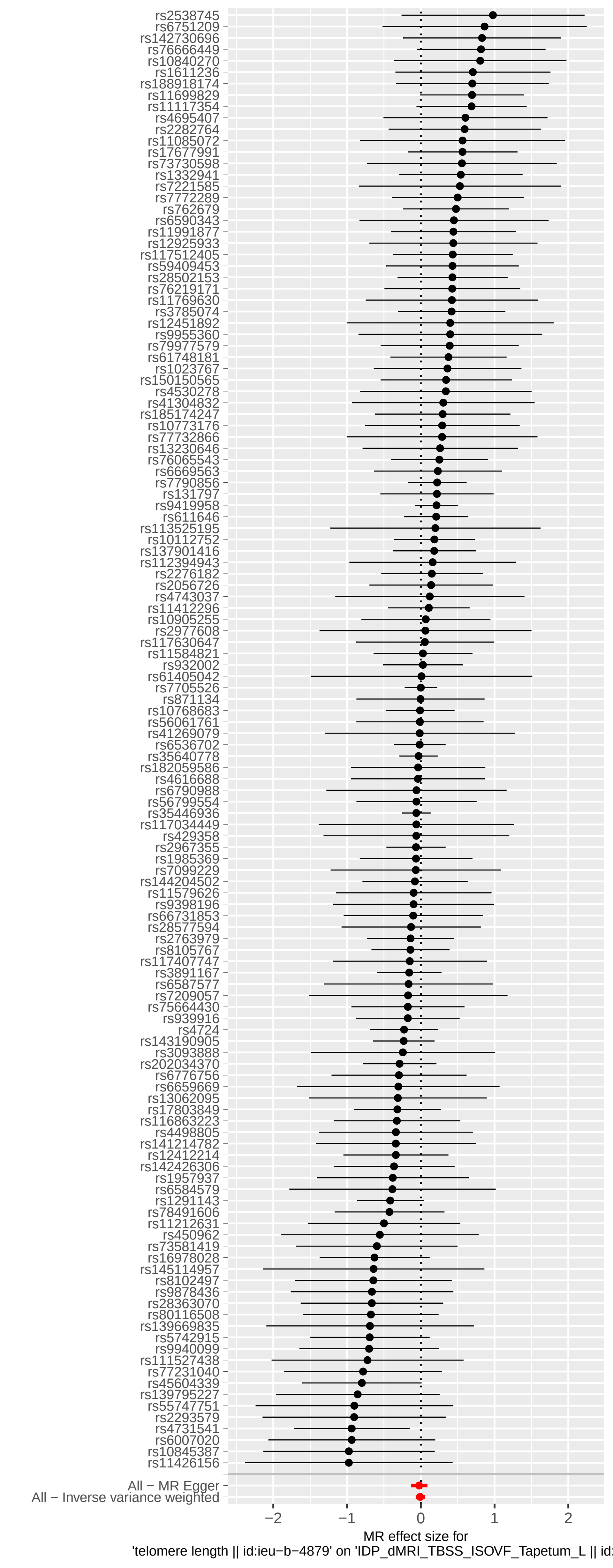
rs2538745
rs6751209
rs142730696
rs76666449
rs10840270
rs1611236
rs188918174
rs11699829
rs11117354
rs4695407
rs2282764
rs11085072
rs17677991
rs73730598
rs1332941
rs7221585
rs7772289
rs762679
rs6590343
rs11991877
rs12925933
rs117512405
rs59409453
rs28502153
rs76219171
rs11769630
rs3785074
rs12451892
rs9955360
rs79977579
rs61748181
rs1023767
rs150150565
rs4530278
rs41304832
rs185174247
rs10773176
rs77732866
rs13230646
rs76065543
rs6669563
rs7790856
rs131797
rs9419958
rs611646
rs113525195
rs10112752
rs137901416
rs112394943
rs2276182
rs2056726
rs4743037
rs11412296
rs10905255
rs2977608
rs117630647
rs11584821
rs932002
rs61405042
rs7705526
rs871134
rs10768683
rs56061761
rs41269079
rs6536702
rs35640778
rs182059586
rs4616688
rs6790988
rs56799554
rs35446936
rs117034449
rs429358
rs2967355
rs1985369
rs7099229
rs144204502
rs11579626
rs9398196
rs66731853
rs28577594
rs2763979
rs8105767
rs117407747
rs3891167
rs6587577
rs7209057
rs75664430
rs939916
rs4724
rs143190905
rs3093888
rs202034370
rs6776756
rs6659669
rs13062095
rs17803849
rs116863223
rs4498805
rs141214782
rs12412214
rs142426306
rs1957937
rs6584579
rs1291143
rs78491606
rs11212631
rs450962
rs73581419
rs16978028
rs145114957
rs8102497
rs9878436
rs28363070
rs80116508
rs139669835
rs5742915
rs9940099
rs111527438
rs77231040
rs45604339
rs139795227
rs55747751
rs2293579
rs4731541
rs6007020
rs10845387
rs11426156
All − MR Egger
All − Inverse variance weighted
−2
−1
0
1
2
MR effect size for
'telomere length || id:ieu−b−4879' on 'IDP_dMRI_TBSS_ISOVF_Tapetum_L || id:
